# Chemical Composition, Antioxidant, Antimicrobial, and Phytotoxic Potential of *Eucalyptus grandis × E. urophylla* Leaves Essential Oils

**DOI:** 10.3390/molecules26051450

**Published:** 2021-03-07

**Authors:** Lijun Zhou, Jiajia Li, Qingbo Kong, Siyuan Luo, Jie Wang, Shiling Feng, Ming Yuan, Tao Chen, Shu Yuan, Chunbang Ding

**Affiliations:** 1 College of Life Science, Sichuan Agricultural University, Ya’an 625014, China; zhouzhou124@126.com (L.Z.); Ljiajia1118@163.com (J.L.); KQB666666@163.com (Q.K.); luosiyuan1998@163.com (S.L.); wj16789@163.com (J.W.); fengshilin@outlook.com (S.F.); yuanming@sicau.edu.cn (M.Y.); chentao293@163.com (T.C.); 2 College of Resources, Sichuan Agricultural University, Chen’du 610000, China; roundtree318@hotmail.com

**Keywords:** *Eucalyptus* oil, antioxidant, antimicrobial, phytotoxic activity

## Abstract

*Eucalyptus grandis × E. urophylla* was a unique hybridization in China. However, the chemical and pharmacological properties were rarely reported. Therefore, in this work, we used a steam distillation method to obtain essential oils from leaves of *E. grandis × E. urophylla*, and further evaluated the antioxidant, antimicrobial, and phytotoxic potential of the essential oil. Gas chromatography mass spectrometry (GC-MS) was applied to investigate the chemical composition of *E. grandis × E. urophylla* essential oil (EEO) and the results showed that the main components of EEO were monoterpenes followed by sesquiterpenes. Among them, α-pinene accounted about 17.02%. EEO could also well scavenge 1,1-diphenyl-2-picrylhydrazyl (DPPH) and 2, 2’-azino-bis(3-ethylbenzothiazoline-6-sulfonic acid) (ABTS) free radicals showing a good free radical clearance ability. In addition, EEO efficiently inhibited the growth of six kinds of bacteria as well as seven kinds of plant pathogens, especially *Salmonella typhimurium* and *Colletotrichum gloeosporioides*. Moreover, the seedling germination of *Raphanus sativus*, *Lactuca sativa*, *Lolium perenne*, and *Bidens pilosa* was significantly suppressed by EEO, thus, indicating essential oils from *eucalyptus* possessed an excellent phytotoxic activity. This study may give a better understanding on EEO and provide a pharmacological activities analysis contributing to the further research of EEO as a functional drug in agronomic and cosmetic industries.

## 1. Introduction

As well known, there were plenty of environmental issues and public health issues for the abuse of pesticides. Traditional agrochemical had a great risk of pesticide/bacterial resistance, environmental pollution, and human health harm. According to Agrow’s report, the total output value of the world agrochemical market had reached USD $31–35 billion in 2007 [1]. Therefore, looking for biopesticide with high efficiency and low toxicity was important.

*Eucalyptus* was the conventional name of *Eucalyptus* of the genus *Myrtaceae* family. It was native to Australia and was one of the most widely cultivated genera in the world [2]. *Eucalyptus* had been widely used in plywood, pulp, and solid wood production, as well its essential oil from leaves had good biological activities [3]. Essential oils from *E. oleosa* [4], *E. globulus* [5], *E. largiflorens* [6], and *E. citriodora* [7] had been proven to possess strong antibacterial activity and widely applied in the field of medicine, food, and the chemical industry. Further research found that *Eucalyptus* essential oil and its components also exhibited the characteristics of herbicides, insecticides, and acaricide [8,9,10]. However, so far, few systematic studies were conducted to focus on the bioactivity of *E. grandis × E. urophylla* essential oil (EEO).

*E. grandis × E. urophylla* was a successful hybrid of *Eucalyptus* by controlled pollination. The advantage of hybridization was remarkable with the characteristics of fast growth and strong stress resistance. Therefore, *E. grandis × E. urophylla* had become the leading variety of artificial afforestation of *Eucalyptus* in China. However, currently, the research studies on *E. grandis × E. urophylla* were mainly focused on the cultivation and wood processing while a few studies were conducted to investigate the active components. The leaves and branches of *E. grandis × E. urophylla* were luxuriant and were proved to be rich in essential oil. However, most of them were abandoned and burned without sufficient utilization.

Therefore, the essential oil from the leaves of *E. grandis × E. urophylla* was extracted by steam distillation, and its composition was investigated by GC-MS. Moreover, the biological activity potential of EEO has been evaluated firstly and systemically. The antioxidant activity was evaluated by determining the scavenging ability on free radicals. In addition, the antibacterial activity was assessed by measuring the inhibition zones, the minimum inhibitory concentration (MIC), and minimal bactericidal concentration (MBC). The antifungal capacity and phytotoxic activity on several weed seeds were also rated in the expectation that the essential oil will be beneficial for the fields of food, medicine, and botanical pesticides.

## 2. Results and Discussion

### 2.1. The Composition of EEO

The yield of essential oil is 0.7% (*w*/*w*, relative to the dry weight) with a color of light yellow and a persistent smell. The components of EEO were qualitatively and quantitatively analyzed by GC-MS, and the results were shown in Table 1. Twenty-seven compounds were identified, corresponding to 97.42% of the total essential oil. Monoterpenes were dominant in *E. grandis × E. urophylla* essential oil (EEO) (67.34%), of which the oxygenated monoterpenes have the most important proportions (32.45%). In addition, sesquiterpene (30.92%) is the main component of EEO. α-pinene (17.02%), α-terpineol (13.63%), aromadendrene (11.08%), d-limonene and (8.47%), and endo-borneol (7.77%) are the major compounds for EEO.

Eucalyptol is the essential component of most *Eucalyptus* plants. However, the main components of essential oil we had obtained were different from those of other varieties, even the hybrid parents. Elaissi analyzed the composition of 20 kinds of *Eucalyptus* essential oils in Northern and Northwestern Tunisia and found oxygen-containing monoterpenoids were the main content of these *Eucalyptus* oil while the highest compound in *E. grandis* oil was 1,8-cineole (15.9%) and p-cymene (11.1%) [11]. Lucia et al. revealed that *E. grandis’*s major components were α-pinene (52.71%) and 1,8-cineole (18.38%) in Argentina [12]. γ-terpinene was the main component of the *E.urophylla* oil [13]. Those *Eucalyptus* distributed in different regions. Hence, varieties and regions may affect the chemical compositions in oils from *Eucalyptus*. In addition, a remarkable difference was found between our work and that of other regions or extraction methods. Liu et al. found that the main constituents of EEO collected in Hainan (China) were alloocimene (43.22%) and α-pinene (13.63%) [14]. The principal constituents of EEO were α-pinene (24.78%) and 1,8-cineole (45.57%), which was collected from three-year-old leaves and was extracted for 8 h at 80 °C by a supercritical fluid extraction (SFE) system using CO_2_ [15]. But 1,8-cineole content of EEO in our study was extremely low at only 1.35%. This may be explained by 1,8-cineole, α-pinene, limonene, and α-terpineol transformed from the same substrate [16]. Different extraction methods led to a substrate conversion to the other three compositions. Therefore, the difference between our study and previous studies may be due to various differences in variety, genetic differences caused by generations and hybridizations, growing environment, extraction method, and even growing years and season of harvesting.

### 2.2. The Antioxidant Abilities of EEO In Vitro

**Figure 1:**
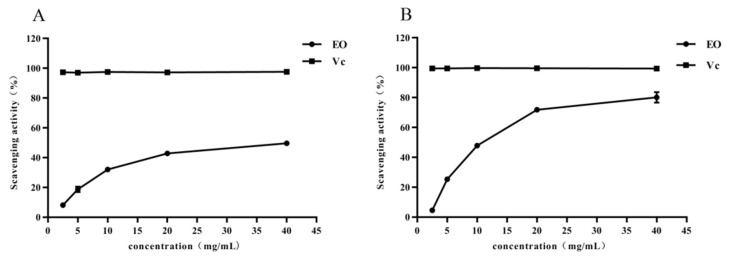
Antioxidant abilities of EEO leaves essential oils, (**A**) DPPH radical scavenging ability and (**B**)ABTS radical scavenging ability.

As shown in Figure 1, EEO exhibited certain antioxidant ability in vitro. EEO could well scavenge 1,1-diphenyl-2-picrylhydrazyl (DPPH) free radicals with a maximum scavenging rate of 50% (Figure 1A). EEO showed an IC_50_ value of 42.5 mg/mL according to our DPPH assay and was powerful than *E. oleosa* essential oil (IC_50_ value of 52.8 mg/mL) [17]. EEO also effectively cleared 2, 2’-azino-bis(3-ethylbenzothiazoline-6-sulfonic acid) (ABTS) free radicals, the maximal eliminating ratio was 80%, and the IC_50_ value was 7.4 mg/mL (Figure 1B). The ABTS activity obtained for EEO (IC_50_ value of 7.4 mg/mL) was also more powerful than the oil of *E. salubris* (IC_50_ value of 273.2 ± 4.1 mg/mL). ABTS radical scavenging ability of EEO was better than its DPPH radical scavenging ability. Comparing the results of the ABTS assay to those of the DPPH one, we could deduce that the ABTS assay generally presented more activity. It could be inferred from the above results that the ABTS assay usually shows more activity than those of the DPPH one. This result may be because ABTS was in aqueous solution and is cationic radical while DPPH in methanol solvent was neutrally radical. Although *Eucalyptus* leaves essential oil showed certain antioxidant capacity, it was still far from the commonly used antioxidant vitamin C (Vc).

It was reported that the antioxidant activity of the *Eucalyptus* was related to the monoterpenes hydrocarbons [17]. *E. citriodora* leaves contain monoterpenoid-rich oil like citronellal (60.66%), exhibiting strong antioxidant activity [18]. *Eucalyptus* essential oil was composed mainly of oxygenated ones (67.8%), monoterpenes (12.2%), and sesquiterpene hydrocarbons (4.9%), which had a moderate activity compared to phenolics and Vc [19]. The percentage of monoterpenes hydrocarbons was 22.8%, sesquiterpene hydrocarbons was 16.8%, and oxygenated monoterpenes was 35.2%. Additionally, a component of 1,8-cineole in the essential oil leaves had been reported to possess potent antioxidant activity [20]. Limonene had remarkable antioxidant activity [21,22], but it was also reported that limonene showed a weak or null effect as a scavenger of DPPH and ABTS radicals [23]. EEO contained a lower level of 1,8-cineole (Table 1), which might be the cause of the moderate antioxidant activity of EEO.

### 2.3. The Antibacterial Activity of EEO

As shown in Table 2, EEO exhibited a great inhibition ability on the six tested-strains. The diameter of the inhibition zone for each bacterium was between 14.33–18.11 mm. The inhibition zone against *S. typhimurium* was the largest (18.11 ± 0.19 mm) while the inhibition zone on *B. subtilis* was the smallest (14.33 ± 0.33 mm). The minimum inhibitory concentration (MIC) value of EEO on strains ranged from 0.023 to 0.091 mg/mL and all the MBC values were 10 mg/mL. A small value of MIC meant an excellent antibacterial effect. Hence, the outcomes demonstrated EEO possessed a strong bacteriostatic ability and the activity against Gram-negative bacteria was higher than against Gram-positive bacteria.

Numerous studies demonstrated that essential oil extracted from *Eucalyptus* leaves had been proven to possess a significant inhibitory effect on bacteria. In addition, 1,8-cineole was considered important for their antibacterial effects. *E. globulus* essential oil had a rather remarkable antimicrobial activity (MIC = 0.09–3.13 mg/mL), especially against *Streptococcus pyogenes* and *Escherichia coli*, and most of which were 1,8-cineole (85.82%) and α-pinene (7.16%) [24]. Essential oils from *E. globulus* and *E. urograndis* could be used to inhibit planktonic cells and biofilm of *S. mutans* with inhibition zones ranging from 14.7 ± 1.2 mm to 35.3 ± 0.3 mm. The inhibiting effect was related to 1,8-cineole [2]. 1,8-cineole was the major compound in the seven kinds of *Eucalyptus* essential oil (49.07 to 83.59%) and their diameter of the inhibition zone was 10 to 29 mm, especially the essential oils from *E. maideni*, *E. astrengens*, *E. cinerea*, and *E. bicostata* that showed the highest antibacterial activity against *Listeria ivanovii* and *Bacillus cereus* [25]. Although some studies showed 1,8-cineole offered a positive effect against bacteria, a few studies showed there is no certain relationship yet. Cimanga concluded no correlation between the content of major constituents such as 1,8-cineole, α-pinene, and the antibacterial activity [26]. The oils of *E. globulus*, *E. radiata*, and *E. citriodora* mainly containing 1,8-cineole (more than 82.66%) were hardly against Gram-negative bacteria [27]. In this present work, the content of 1,8-cineole in EEO was very low, while EEO had a strong bacteriostatic effect. Hence, 1,8-cineole could not be the vital composition. It can be seen that other components play a key role in a bacteriostatic effect. The antimicrobial activity of α-terpineol, terpinene-4-ol, and α-pinene had been reported [28,29,30]. Therefore, the antibacterial activity of EEO may be due to these terpenes and the cooperative action of them.

Drug resistance of bacteria was a big problem in modern agriculture and the food industry. It was urgent to develop new bioactive molecules from natural sources with lower side effects as a substitute for antibiotics [31]. Some plant essential oil had complex chemical compositions that make it difficult for bacteria to develop resistance and had become an important source of novel antibiotics against drug-resistant bacteria [32]. Freitas conclude that α-pinene had antibacterial and antibiotic-modulating activities by enhancing the activity of norfloxacin and gentamicin [33]. Therefore, EEO as a natural product had good antibacterial activity that might be used for improved agents of antibiotics.

### 2.4. The Antifungal Activity of EEO

As shown in Table 3, EEO could completely inhibit the seven strains when the concentration reached 40 mg/mL. When the concentration was 20 mg/mL, EEO could completely suppress the growth of six strains except *F. graminearum* with an inhibitory rate of 61.52 ± 1.84%. *E. grandis × E. urophylla* essential oil (EEO) still exhibited a good inhibitory effect on plant pathogens (14.52%–100%) with the concentration below 10 mg/mL, especially *C. gloeosporioides* followed by *B. cinerea*. Liu et al. found terpenes played a key role in the inhibitory effects of *E. grandis × E. urophylla* oil on *Fusarium oxysporum*, *Pyriculerie grisea*, *Glorosprium musarum*, and *Phytophthora capsici* [14]. Zhou et al. found the essential oil extracted by supercritical CO_2_ method from *E. grandis × E. urophylla* leaves exhibited a great antifungal effect on 11 plant pathogenic fungi, such as *Fusarium moniliforme*, *Fusarium graminearum*, and *Magnaporthe grisea* [34]. These were in agreement with the results of our paper that EEO had broad-spectrum inhibitory effects to many fungi so that it could become a potential for biological pesticide.

Anthracnose was an infection of *Colletotrichum gloeosporioides* and was a significant pathogen implicated in many diseases of fruits and crops in many countries and regions around the world. Essential oil extracted from *Eucalyptus* leaves have been proven to possess a significant inhibitory effect on a variety of plant pathogens including *C. gloeosporioides* [35]. Essential oils from three *Eucalyptus* (*E. camaldulensis* Dehn, *E. globulus* Labill, and *E. tereticornis* Smith) in Colombia were also proven to have a good inhibition effect on *C. gloeosporioides* when the concentration was higher than 2.5 mg/mL [36], which were in agreement with our results. Chang et al. found the fungicidal activity of limonene, α and β-pinene against *F. solani* and *C. gloeosporioides* [37]. Terpene-4-ol and α-terpineol could effectively inhibit the mycelium growth and spore germination [38]. α-terpineol had been reported to have antimicrobial properties and to enhance the permeability of skin to lipid-soluble compounds widely used in aromatherapy and folk medicine [39,40]. The antifungal activity of globulol against the fungus *Botrytis cinerea* had been determined [41]. *E. camaldulensis* oil presented higher antifungal activity over *C. gloeosporioide* than *E. globulus* oil and it had a higher level of globulol than *E. globulus* oil [36]. EEO contained a certain level of α-pinene, globulol, and α-terpineol. Therefore, we speculated that those components play a significant role in antifungal activity of EEO.

### 2.5. The Phytotoxic Activity of EEO

**Figure 2:**
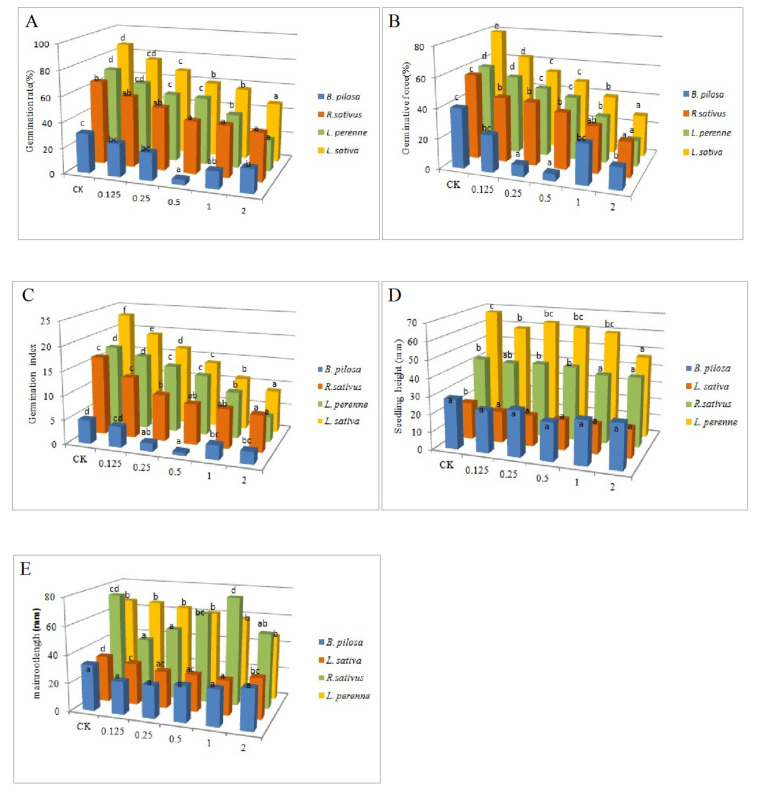
Effects of *E. grandis × E. urophylla* essential oil (EEO) at different mass concentrations on seed (**A**) germination rate, (**B**) germination force, (**C**) germination index, (**D**) seedling height and (**E**) the growth of roots. CK means blank control (1% Tween 80 solution). Error bars correspond to the standard error of each mean. For each observation day, means followed by the same letter (a–d) are not statistically different ((Tukey’s test *p ≤ 0.05*).

Statistical significance in Figure 2 was evaluated by one-way ANOVA, which was followed by the Tukey-Kramer multiple comparison test. The results show that EEO had certain phytotoxic potential, especially for four kinds of tested seeds. The germination rate, germination force, and germination index of the three seeds (*R. sativus*, *L. sativa*, and *L. perenne*) decreased with the increase of the concentration of EEO (0.125–2 mg/mL) among which the seed of *L. perenne* was the most sensitive to EEO. The inhibitory effect on seed of *B. pilosa* increased first and then decreased with the increase of concentration of EEO. It was the strongest when the concentration of EEO was 0.5 mg/mL (Figure 2A–C). EEO showed a certain inhibition in seedling height (Figure 2D). The length of the main root of *R. sativus* and *L. perenne* were significantly suppressed after treatment with a different concentration of EEO. However, EEO showed no significant effect on the main root of *R. sativus* and *B. pilosa*. All main root lengths of four seeds were shorter than that of the blank group, indicating EEO could restrain the growth of roots (Figure 2E). EEO has good phytotoxicity, which may slow down the use of traditional herbicides.

Phytotoxic substances had high sensitivity and selectivity, and the sensitivity of phytotoxic substances to different receptor plant phytotoxic effects was different [42]. *Eucalyptus* essential oil had a certain phytotoxicity. *E. camaldulensis* oil significantly reduced *E. bonariensis* seed germination [43]. Li et al. found that essential oil from 17 cultivars *Eucalyptus* strongly inhibits germination and growth of *L. perenne* [44]. Essential oils of *E. salubris*, *E. spathulata*, *E. brockwayii*, and *E. dundasii* also had stronger inhibitory effects on germination and seedling growth of silverleaf nightshade [45].

Abrahim et al. found that α-pinene strongly inhibited mitochondrial ATP production of maize seedlings [46]. Singh et al. demonstrated that α-pinene inhibited early root growth and caused oxidative damage in root tissue of five test species, including *Cassia occidentalis, Amaranthus viridis*, *Triticum aestivum*, *Pisum sativum*, and *Cicer arietinum* [47]. The germination and radical elongation of *R. sativus* and *Lepidium sativum* were inhibited by α-pinene [48]. Terpineol had been also recognized as phytotoxic [49]. Although some of the components have a good phytotoxic effect, the phytotoxicity of EEO was likely to owe to the synergistic action of different monoterpenes. Whether the observed phytotoxicity effect of EEO was attributable to a single compound or to the synergistic effects of several compounds need to be investigated further.

## 3. Materials and Methods 

### 3.1. Materials and Chemicals

In May 2019, fresh *Eucalyptus* leaves were collected and dried naturally in Yuping Town, Hongya County, Meishan City, Sichuan Province, China (29°52′36.52″ N, 103°29′17.51″ N). The dried leaves were crushed and sifted, and then stored in a refrigerator at 4 °C for the following experiments. 1,1-diphenyl-2-picrylhydrazyl (DPPH) was purchased from Sigma Chemical Co. (St. Louis, MO, USA). Anhydrous sodium sulfate, ethanol, glucose, sodium chloride, and acetic acid were purchased from the Chengdu Kelong Chemical Factory (Chengdu, China). Tryptone and yeast extract were obtained from OXOID (Basingstoke, UK). 2, 2’-azino-bis(3-ethylbenzothiazoline-6-sulfonic acid) (ABTS) kits were purchased from Nanjing Jiancheng Bioengineering Institute (Nanjing, China). All chemicals above were of an analytical grade.

### 3.2. Extraction of Essential Oil

The powder of *Eucalyptus* leaves (sieved with 60 mesh) was loaded into a steam distillation device (2000 mL) and extracted in an electric heating-jacket for 6 hours at 100 °C with distilled water to a solvent to sample the ratio of 15:1 mL/g. After stratification, the water was removed with anhydrous sodium sulfate. Then the essential oil was collected and stored in a brown bottle at 4 °C.

### 3.3. Gas Chromatography-Mass Spectrometry (GC-MS) Analysis of EEO

The EEO was diluted to 10 μL/mL by n-hexane and n-hexane was used as blank control. Gas chromatographic conditions: Gas Chromatography Mass Spectrometer (Agilent, 7890B-5977A, Agilent Technologies Inc., Santa Clara, CA, USA), HP-5MS column (30 cm × 0.25 mm × 0.25 μm), using the temperature program, hold at 40 °C for 5 min, and then rise to 100 °C at the speed of 10 °C/min, keeping at 5 min. Raise the temperature to 160 °C at the speed of 10 °C/min. Then keep for 5 min. Subsequently, the temperature was raised from 10 °C/min to 280 °C to keep at 5 min. Helium was used as the carrier gas and the injection volume was 1 μL with no shunt. The injection port temperature was 260 °C, and the interface temperature was 220 °C. Mass spectrometry conditions: electron bombardment (EI) ion source, electron energy was 70 eV, and electron multiplier voltage was 1.5 kV [50]. Mass scanning ranges from 33 to 600 fulling scanning and injection volume was 1 μL. The EEO qualitative identification was based on a comparison of mass spectrometry with standards recorded by the National Institute of Standards and Technology (NIST) libraries. In addition, the retention index (RI) was given by the literature. While quantitative analysis of the individual component was carried out by a peak area normalization measurement, the relative content of each component was calculated by the percentage of each peak area [51].

### 3.4. In-Vitro Antioxidant Abilities

#### 3.4.1. Determination of DPPH-Free Radical Scavenging Ability

DPPH-free radical scavenging assay was conducted according to the previous method reported by Luís et al. [52] with some small modifications. EEO samples were diluted to different concentrations (2.5, 5, 10, 20, and 40 mg/mL) by ethanol. In addition, 30 μL of the sample solution and 170 μL of the DPPH ethanol solution (0.4 mM) were added to the 96-well plate for 15 min in the dark at room temperature (24–26 °C). Then the absorbance was determined at 517 nm. In this study, absolute ethanol and ascorbic acid (Vc) were used as a blank control and a positive control, respectively. The scavenging rate of a DPPH-free radical was calculated according to the following formula (Equation (1)):

DPPH radical scavenging rate (%) = 1 − A1/A0 × 100%(1)

where the A1 was the absorbance of the sample and DPPH solution and A0 was the absorbance of a blank control.

#### 3.4.2. Evaluation of ABTS-Free Radical Scavenging Ability

The ABTS radical scavenging ability of EEO was determined according to the method of the ABTS assay kit. Furthermore, 190 μL of ABTS working fluid was mixed with 10 μL of different concentrations of EEO (2.5, 5, 10, 20, and 40 mg/mL) and then incubated at room temperature (24–26 °C) for 6 min. Ethanol was used as the blank control and ascorbic acid (Vc) solution was the positive control. The absorbance of each mixture was measured at 734 nm. The scavenging ability of ABTS-free radicals was calculated according to the following formula (Equation (2)):

ABTS radical scavenging rate (%) = 1 − A1/A0 × 100%(2)

where the A1 was the absorbance of the sample and ABTS solution and A0 was the absorbance of a blank control.

### 3.5. Antimicrobial Assays

#### 3.5.1. Microbial Strains for Antimicrobial Screening

*Escherichia coli*, *Pseudomonas aeruginosa*, *Staphyloccocus aureus*, *Bacillus cereus*, *Bacillus subtilis*, and *Salmonella typhimurium* strains were obtained from College of Life Sciences, Sichuan Agricultural University. All bacteria strains were cultivated in LB medium. *Trichoderma longibrachiatum*, *Colletotrichum gloeosporioides*, *Fusarium graminearum (Schw.) Petch*, *Botrytis cinerea*, *Fusarium oxyspoyum f.sp.niveu*, and *Colletotrichum acutatum* were given by the College of Agronomy, Sichuan Agricultural University.

#### 3.5.2. Determination of Inhibition Zones

The antibacterial activity of EEO was evaluated by the paper-disc agar diffusion method [11]. A round filter paper with a diameter of 6 mm was made with a hole punch and sterilized. In total, 400 μL of bacterial solution (10^6^ CFU) evenly spread on the luria bertani medium (LB) plate. Four filter papers were placed on the plate after the solution was dried. A total of 5 μL of EEO of different concentrations was dripped on the paper and 1 mg/mL ampicillin sodium was used as a positive control. The plate was then incubated at 37 °C for 24 h. The diameter of the inhibition zone was measured and expressed in millimeters. The evaluation of the bacteriostatic effect was usually based on the width of the inhibition zone diameter (izd), which is not sensitive for a diameter of ≤8 mm, sensitive at a diameter of 8–14 mm, very sensitive at a diameter of 14–20 mm, and extremely sensitive for a diameter of ≥20 mm [11].

#### 3.5.3. Determination of the Minimum Inhibitory Concentration (MIC) and Minimum Bactericidal Concentration (MBC)

Determination of minimum inhibitory concentrations (MIC) and minimum bacterium concentration (MBC) was conducted according to the previous method [6]. Briefly, EEO was diluted to 10 mg/mL with LB liquid medium and filtered by 0.22 μm of the filter membrane. EEO was added to 96-well plate and then diluted from the first well to another by the dilution method. Subsequently, 100 μL of bacterial solution (concentration 10^6^ CFU) was added into each well (containing 100 μL of EEO). Ampicillin sodium was added as the positive control and the blank control was in the absence of EEO. Then, the plate was placed at 37 °C for 20 h before reading the absorbance at 600 nm. The MIC was identified as the absorbance of the test group was significantly lower than that of the blank control. In total, 100 μL of solution was absorbed from the wells where there was no clear bacterial growth and smeared on LB medium. The colony number was calculated after the plates were cultured at 37 °C for 24 h. MBC was determined as the lowest essential oil concentration with the colony number less than 1.

#### 3.5.4. Antifungal Assay

The antifungal activity of EEO was evaluated by the growth rate method [34]. EEO resolved by 1% Tween 80 was added to PDA medium to prepare an essential oil PDA culture plate with final concentration at 2.5, 5, 10, 20, and 40 mg/mL. The PDA culture plate prepared by 1% Tween 80 solution was used as a blank control. The strains were punched into 6-mm hyphal pieces. The inoculation shovel transferred the prepared piece on the medium as the mycelial side was down. Then, the medium was incubated at 25 °C. The mycelial growth was observed every day and the diameter of each culture plate was measured with a Vernier caliper (the average value was measured by centimeter by the cross method for three times) until the culture plate of the blank control was full of mycelium. The inhibitory effect of essential oil was evaluated by the inhibition rate of the day before the blank control board was full (Equation (3)):

Antifungal activity (%) = (A0 − A1)/(A0 − 6)(3)

where the A0 was the diameter of the blank control and A1 was the diameter of the test group.

### 3.6. Phytotoxic Activity

According to the previous bioassay protocol, Reference [45] was adopted with slight modifications. The seeds of *Raphanus sativus*, *Lactuca sativa*, *Lolium perenne*, and *Bidens pilosa* were purchased from the seed shop. Sterilized filter papers were put in a sterilized 9-cm diameter petri dishes with 4 mL of different concentrations of EEO and 1% Tween 80 solution as the blank control. Then, 30 seeds of radish, lettuce, ryegrass, and bidens were placed on the filter paper (Chengdu Kelong Chemical Factory, Chengdu, China) in order. Then, the flasks were placed at 22 ± 1 °C for incubation. With the germ root or hypocotyl breaking through the seed coat of 1–2 mm as the seed germination standard, the number of germination of the recipient plant seeds was observed and recorded daily. The statistics were calculated continuously for 7 days to calculate the final germination rate (Equation (4)), germination force (an index used to evaluate the germination speed and uniformity of seeds) (Equation (5)), and germination index (Equation (6)). On the seventh day, the seedling height and main root length of the seedlings (the length from the end to the end of the root) were measured using a vernier caliper.

Germination rate = (A1/A0) × 100%(4)

where the A1 was the total number of germinated seeds and A0 was the total number of tested seeds.

Germination force = B1/A0(5)

where the B1 was a number of seeds germinated at the peak of germination and A0 was the total number of tested seeds.

Germination index = ∑(Gt/Dt)(6)

where the Gt was the number of germinations on the t day and Dt was the number of corresponding germination days.

### 3.7. Statistical Analysis

All the experimental groups were conducted in triplicates. The outcomes were expressed as mean ± SD. Graphpad Prism 6.0 (GraphPad Software, Inc., LaJolla, CA, USA) was used for statistical analysis and making graphic pictures. The significance test was analyzed by SPSS 19.0 using the ANOVA method and the significance level was considered as *p* < 0.05.

## 4. Conclusions

GC-MS analysis showed that EEO was mainly composed of oxygen-containing monolithic compounds. In addition, the essential oil showed a certain ability on scavenging free radicals and could be used as a potential antioxidant. EEO possessed a strong antibacterial activity, which could effectively inhibit a variety of bacteria and plant pathogenic fungi, especially *S. typhimurium* and *C. gloeosporioides*, indicating EEO had a broad application prospects in food and pharmaceutical products. Essential oil from *E. grandis × E. urophylla* had a clear phytotoxic effect. The significant antibacterial as well as phytotoxic capacities were conducive to more research studies and development in the direction of biological pesticides. Nevertheless, further investigations will be carried out to evaluate the antibacterial mechanism, as the essential oil could be applied to food and pharmaceutical products.

## Figures and Tables

**Table 1:** Chemical compositions of EEO.

Number	Retention Time (min)	Retention Indices (RI)	Compounds	Percentage (%)
1	9.564	931	α-pinene	17.02
2	9.906	943	camphene	2.83
3	11.466	1025	o-cymenep-cymene	5.76
4	11.605	1030	eucalyptol	1.35
5	11.703	1034	trans-β-ocimene	0.31
6	12.184	1047	γ-terpinene	0.51
7	13.677	1100	fenchol	4.68
8	14.032	1120	α-campholenal	2.25
9	15.088	1146	isoborneol	0.25
10	15.519	1148	endo-borneol	7.77
11	15.881	1161	terpinen-4-ol	2.04
12	16.548	1172	α-terpineol	13.63
13	17.646	1228	Isobornyl formate	1.37
14	18.01	1299	myrtenyl acetate	0.37
15	21.04	1373	isoledene	0.25
16	21.91	1424	β-caryophyllene	2.63
17	22.296	1439	aromadendrene	11.08
18	22.493	1456	α-humulene	0.36
19	22.872	1494	γ-muurolene	0.34
20	24.663	1530	epiglobulol	2.86
21	25.77	1597	ledol	0.65
22	26.755	1628	τ-cadinol	0.54
23	27.188	1641	α-cadinol	0.56

**Table 2:** Antibacterial activity of EEO.

Parameters	*E. coli* (−)	*B. subtilis* (+)	*P. aeruginosa* (−)	*S. aureus* (+)	*S. typhimurium* (−)	*B. cereus* (+)
Inhibition zone (mm)	15.22 ± 0.38	14.33 ± 0.33	15.17 ± 0.24	15.11 ± 0.84	18.11 ± 0.19	15.78 ± 0.38
MIC (mg/mL)	0.091	0.091	0.023	0.045	0.023	0.045
MBC (mg/mL)	10	10	10	10	10	10

Notes: (+) meant Gram-positive bacteria and (−) meant Gram-negative bacteria.

**Table 3:** Antifungal activity of EEO.

	Inhibition Rate (%)	The Concentration of EEO (mg/mL)				
Strains		2.5	5	10	20	40
*Trichoderma longibrachiatum*		15.96 ± 2.42	47.15 ± 5.67	90.91 ± 5.82	100	100
*Botrytis cinerea*		16.74 ± 1.98	68.44 ± 1.71	100	100	100
*Colletotrichum acutatum*		14.44 ± 2.77	40.45 ± 2.06	78.58 ± 1.30	100	100
*Colletotrichum gloeosporioides*		41.29 ± 1.91	82.57 ± 4.08	100	100	100
*Fusarium oxyspoyum*		34.95 ± 2.20	44.46 ± 1.62	78.38 ± 1.40	100	100
*Fusarium graminearum*		16.97 ± 3.85	32.88 ± 2.33	45.49 ± 1.01	61.52 ± 1.84	100

## Data Availability

Data is contained within the article.

## Sample Availability

Samples of the *E. grandis × E. urophylla* essential oil are available from the authors.
